# Changes in biomarkers after 180 days of tobacco heating product use: a randomised trial

**DOI:** 10.1007/s11739-021-02798-6

**Published:** 2021-07-01

**Authors:** Nathan Gale, Michael McEwan, Oscar M. Camacho, George Hardie, Christopher J. Proctor, James Murphy

**Affiliations:** 1 British American Tobacco (Investments) Limited, Research and Development, Regents Park Road, Southampton, SO15 8TL UK; 2 DoctorProctorScience Limited, 157 Cavendish Meads, Sunninghill, Ascot, SL5 9TG UK; 3 R. J. Reynolds Tobacco Company, 401 N Main Street, Winston-Salem, NC27101 USA

**Keywords:** Cigarette smoking, Tobacco heating product, Biomarkers of exposure, Biomarkers of potential harm, Modified risk tobacco product

## Abstract

The aim of this study was to investigate whether biomarkers of exposure (BoE) and potential harm (BoPH) are modified when smokers switch from smoking cigarettes to exclusive use of a tobacco heating product (THP) in an ambulatory setting. Participants in this randomised, controlled study were healthy volunteer smokers assigned either to continue smoking or switch to a THP, and a control group of smokers who abstained from cigarette smoking. Various BoE and BoPH related to oxidative stress, cardiovascular and respiratory diseases, and cancer were assessed at baseline and up to 180 days. In continuing smokers, BoE and BoPH remained stable between baseline and day 180, while THP users’ levels of most BoE reduced significantly, becoming similar to those in controls abstaining from cigarette smoking. Also at 180 days, significant changes in numerous BoPH, including total 4-(methylnitrosamino)-1-(3-pyridyl)-1-butanol, 8-epi-prostaglandin F2α type III, fractional concentration of exhaled nitric oxide and white blood cell count, were directionally consistent with lessened health impact. Our findings support the notion that the deleterious health impacts of cigarette smoking may be reduced in smokers who completely switch to using THPs.

**Supplementary Information:**

The online version contains supplementary material available at 10.1007/s11739-021-02798-6.

## Introduction

Cigarette smoking is linked to the development of numerous diseases including lung cancer, cardiovascular disease and chronic obstructive pulmonary disease [1]. Smoking-related disease risk is correlated to daily cigarette consumption and the number of years since smoking initiation and is due to inhalational exposure to smoke toxicants that transfer into cigarette smoke during tobacco combustion [1‒6]. While quitting smoking reduces disease risk [1], and large proportions of smokers report wanting to quit smoking and make cessation attempts [6], fewer than one in ten smokers successfully quit smoking annually [7]. For those who are either unwilling or unable to quit smoking, a tobacco harm reduction (THR) approach has been proposed [6]. Fundamentally, THR relies on the proposition that the health burden of smoking at the individual and population levels can be reduced by encouraging smokers to switch to novel nicotine and tobacco products that may support combustible cigarette displacement [8], and while not being risk free would reduce or eliminate exposure to toxicants [8, 9] and potentially reduce smoking-related harms.

Cigarette smoke contains more than 8700 identified chemicals [5], many of which may contribute to disease development [10]. The US Institute of Medicine (IoM) has proposed that the development of potential reduced-exposure products (PREPs) which yield lower emissions of some toxicants compared with conventional cigarettes could be expected to result in reduced toxicant exposure in smokers who completely switch to using them [4, 6]. Aerosols from tobacco heating products (THPs) exhibit lower machine yields of toxicants compared to cigarette smoke [11]. Clinical studies examining smokers who switch to using THPs have demonstrated reductions in exposure, in some cases to a degree approaching or matching that of smoking cessation [12‒14]. Despite these exposure reductions and demonstrations that novel tobacco products may be PREPs, what is not fully established is whether switching to using THPs leads to measurable changes in the health impacts of smoking. One approach to assess the potential health impacts of switching is to measure biomarkers of potential harm (BoPH) [15, 16] in clinical studies involving switching smokers. BoPH assessment has been defined as “measurement of an effect due to exposure; these include early biological effects, alterations in morphology, structure, or function, and clinical symptoms consistent with harm” [15]. Studies utilising BoPH can help determine whether a PREP can be considered a modified risk tobacco product (MRTP) [16] and may form a substantial component of regulatory submissions to regulators such as the US Food and Drug Administration (FDA) when requesting authorization to market a novel product as a MRTP [16, 17].

The aim of this current study is to examine changes in BoE and BoPH in smokers who switch to using a THP relative to those who continue to smoke combustible cigarettes, over a period of 12 months. We have recently reported BoE changes at day 90 of this study [12], and here we report both BoE and BoPH findings up to day 180.

## Methods

### Study design

This was a randomised, controlled, parallel group, open-label, ambulatory clinical study carried out at four sites in the UK (Belfast, London, Leeds and Merthyr Tydfil). Favourable opinion (which is equivalent to Institutional Review Board (IRB) approval) was given by the NHS Health Research Authority, Wales Research Ethics Committee 2 (reference number 17/WA/0212). The study was conducted in compliance with the ethical principles of the Declaration of Helsinki, Good Clinical Practice (International Council for Harmonisation (ICH) E6 Consolidated Guidance, April 1996) and UK laws, including those relating to the protection of participants’ personal data. Written informed consent was obtained from all participants prior to their participation in the study and before undergoing any study procedures, including screening assessments. A full description of the study design and protocol has been published previously [18]. This study is registered with ISRCTN (ISRCTN81075760).

### Participants

During a screening visit, potential participants were assessed. Eligible participants were healthy male or female adult current smokers (self-reported daily smoking of 10–30 non-menthol factory-manufactured or roll-your-own cigarettes for at least 5 consecutive years) or never-smokers aged 23‒55 years. Smoking status was verified using urinary cotinine (> 200 ng/mL) and exhaled breath carbon monoxide (eCO; ≥ 7 ppm). The cotinine cut-off used was based on the ability to discriminate between social/intermittent smoking and regular smoking [19]. Main inclusion criteria were no clinically relevant abnormal findings on physical examination, vital signs assessment, electrocardiogram, clinical laboratory evaluations or lung function tests, and medical history. The main exclusion criteria were refusal of individuals or their partners of childbearing potential to use effective methods of contraception for the duration of the study; females who were pregnant/breastfeeding; blood donation ≥ 400 mL within 12 weeks (males) or 16 weeks (females) prior to study start; acute illness requiring treatment within 4 weeks prior to study start; regular use of any nicotine/tobacco products other than commercially manufactured filter cigarettes and/or roll-your-own cigarettes up to 14 days before screening; use of any medications/substances (other than tobacco) which interfere with the cyclo-oxygenase pathway or are known to be strong inducers or inhibitors of cytochrome P450 enzymes, up to 14 days or five half-lives of the drug prior to study start. Participants who were never-smokers or were planning to quit in the next 12 months could be included but were eligible only for the never-smoker or cessation groups, respectively.

### Study procedures and randomisation

A study design schematic has been published previously [18]. Following screening procedures, smokers completed a tobacco use history questionnaire and the Fagerström Test for Cigarette Dependence (FTCD) [20]. At Visit 1 (baseline), participants underwent safety assessments prior to randomisation. Ambulatory 24-h urine samples and spot blood samples were taken for BoE and BoPH analysis, eCO and fractional concentration of exhaled nitric oxide (FeNO) measurements were made, and spirometry was performed. Smokers not intending to quit were also allowed to try the THP to experience the product to which they might be randomised. Participants could decide whether to continue to participate in the study following this trial.

Randomisation schemes were computer-generated by Covance Clinical Research Unit (Leeds, UK) using a pseudo-randomisation permutation procedure (PROC PLAN procedure in SAS^®^ Version 9.4) for the continue smoking group (Group A) and the switch to THP group (Group B) and provided to the study centres. Randomisation lists were stratified by sex and age categories (23–40 years and 41–55 years). Participants were assigned to groups in blocks of eight, with two participants allocated to Group A and six to Group B within each block [21]. Participants intending to quit were assigned without randomisation to the cessation group (Group D), and an attempt was made to achieve a balance by sex and age. Never-smokers were assigned to Group E.

All participants attended the clinic on days 30, 60, 90 and 180 (Visits 2, 3, 4 and 7), at which the same samples were collected as Visit 1. In addition to eCO measurements made at these visits, eCO was also measured on days 120 and 150 (Visits 5 and 6) and values reported here are the mean of these 2 measurements.

All participants received a Research Ethics Committee-approved financial reimbursement for taking part in the study, which was set by the clinical site in accordance with their usual level of stipend for taking part in this type of study and was dependent on the number of procedures each participant underwent. Smokers were reminded of the risks associated with smoking prior to enrolment and informed that they were free to voluntarily quit smoking and/or withdraw from the study at any time. Any participant who decided to quit smoking was directed to appropriate stop smoking services.

Adverse and serious adverse events were monitored throughout the study period by open questioning at each study visit and by encouraging participants to spontaneously report such events by telephone should they occur between study visits. Reported adverse events were recorded in source data and on electronic case report forms and coded according to MedDRA Version 20.0. Adverse events were any medical event, irrespective of being related to the investigation products. Serious adverse events were defined as those resulting in death, threatening to life, requiring hospitalisation/prolongation of hospitalisation, resulting in disability and/or in congenital anomaly or birth defect.

### Investigational products

Participants in Group A were required to purchase their own usual-brand cigarettes. Those in Group B received the glo THP device and Neostick tobacco consumables (British American Tobacco, Southampton, UK) free of charge. These products have been described previously [12, 22]. In brief, the glo THP electronically heats a small tobacco consumable (Neostick) to a temperature of approximately 245 °C. This eliminates the combustion of tobacco but facilitates the release of nicotine in an aerosol which the user inhales [12].

At study visit 1, participants randomised to Group B were provided by clinic staff with the study THP and tobacco consumables (one Neostick being equivalent to one cigarette) equivalent to 150% of their average number of cigarettes consumed per day (CPD) as self-reported at screening, with the possibility of more (up to a total of 200% of original CPD consumption) before visit 2 by visiting the study site. At visits 2–12, product usage was assessed by return of all empty, part-used, and unused packs of THP consumables, and the next allocation of consumables was supplied at 120% of the usage in the previous period, up to the limit of 200% of pre-screening consumption. At visit 13, as well as all empty, part-used and unused packs of THP consumables, participants were asked to return the study THP device, chargers and other accessories supplied for use in this study. The 200% limit was chosen to support naturalistic product use behaviour following switching to THP use due to possible difference in nicotine yield from usual brand cigarettes, but to avoid large increases in the consumption of free tobacco products which has been reported previously in similar studies [23, 24]. Full accountability records for study products (THP device and consumables) were maintained by staff at the clinical site.

Group D participants devised a cessation strategy with the Investigator, which included nicotine replacement therapy (NRT) and/or varenicline provision if requested, alongside cessation counselling.

### Compliance

Participants were instructed of the importance of exclusively using their randomised product (Groups A and B) or of not smoking cigarettes or using nicotine products (Groups D and E) other than NRT (Group D). Participants were asked to report any non-compliance using electronic or paper diaries and were informed that compliance assessments would be conducted at each study visit. Assessment of compliance in Group B was achieved by measuring levels of a haemoglobin adduct of acrylonitrile (*N*-(2-cyanoethyl) valine; CEVal) as a marker of combusted tobacco exposure. Acrylonitrile is found in cigarette smoke but is below the detection limit in the THP emissions and has no common environmental source. Thresholds for CEVal used to deduce compliance were calculated based on a previous study [21, 23].

Use of concomitant medication by study participants was recorded by study site staff. If a prohibited concomitant medication which could affect BoE/BoPH was taken, the participant’s data for the timepoint(s) affected by that concomitant medication were not included in any analyses.

### Biomarkers of exposure

BoE to selected cigarette smoke constituents in 24-h urine collections were measured at baseline and days 30, 60, 90, and 180; this paper reports BoE levels on days 90 and 180. Laboratory analyses of urine and blood BoE were carried out at ABF GmbH (Planegg, Germany). Details of the bioanalytical methods have been published previously [13]. All BoE assessed in this study have been assessed as fit for purpose in cigarette smoke exposure studies using criteria such as the availability of suitable assay techniques, sample stability, reproducibility, differential levels between smokers and non-smokers, and the kinetics of reversibility with either smoking cessation or changes in tobacco product use [25].

BoE measured in 24-h urine samples were total nicotine equivalents (TNeq; nicotine, cotinine, 3-hydroxycotinine and their glucuronide conjugates); total 4-(methylnitrosamino)-1-(3-pyridyl)-1-butanol (NNAL); total N-nitrosonornicotine (NNN); 3-hydroxypropylmercapturic acid (3-HPMA); 3-hydroxy-1-methylpropylmercapturic acid (HMPMA); S-phenylmercapturic acid (S-PMA); monohydroxybutenyl-mercapturic acid (MHBMA); 2-cyanoethylmercapturic acid (CEMA); 4-aminobiphenyl (4-ABP); *o-*Toluidine (*o-*Tol); 2-aminonaphthalene (2-AN); 1-hydroxypyrene (1-OHP); and 2-hydroxyethylmercapturic acid (HEMA). Additionally, eCO in exhaled breath and CEVal in whole blood were measured. The smoke constituent associated with each BoE, and details of the limit of detection and lower and upper limits of quantification for each BoE measured, have been reported previously [12].

### Biomarkers of potential harm

BoPH were assessed in urine (11-dehydrothromboxane B2 [11-dTx B2], 8-epi-Prostaglandin F2a type III [8-Epi-PGF2α type III]), whole blood (white blood cell [WBC] count), plasma (soluble intercellular adhesion molecule-1 [sICAM-1]), serum (high-density lipoprotein [HDL]), and exhaled breath (FeNO). Additionally, forced expiratory volume in 1 s (FEV_1_) was assessed using spirometry. Indications associated with each BoPH have been reported previously [18, 21]. BoPH selection was based on a number of criteria, including association of the BoPH to the risk of developing a smoking-related disease, previously reported differences in BoPH levels between smokers and non-smokers, existence of a dose–response relationship between cigarette consumption and BoPH levels, and reversibility and kinetics after smoking cessation [26]. Furthermore, the selected BoPH have been assessed in prior studies examining the impact of switching from cigarette smoking to using novel nicotine products on individual health markers [27‒29]. While NNAL is generally used as a BoE to the cigarette smoke toxicant 4-(methylnitrosamino)-1-(3-pyridyl)-1-butanone (NNK), it is also considered to be a BoPH for smoking-related lung cancer risk due to its tobacco specificity, its carcinogenicity, and its predictive value for lung cancer risk [30‒33]. Laboratory analyses of urine and blood (whole, plasma, and serum) BoPH were carried out at Celerion (Lincoln, NE, USA) and Covance (Harrogate, UK and Geneva, Switzerland). sICAM-1 was measured using an electrochemiluminescence immunoassay (Meso Scale Diagnostics, Rockville, MD, USA). FeNO was measured using a NIOX VERO™ device (Circassia Ltd, Oxford, UK) and spirometry was measured using a 6600 Compact™ Expert Workstation Spirometer (Vitalograph Ltd, Buckingham, UK). WBC counts were performed using an automated hematology sampling procedure (Covance). HDL was assessed using homogenous enzymatic colorimetry (Roche Diagnostics, Mannheim, Germany). 11-dTx B2 and 8-Epi-PGF2α type III were assessed using gradient ultra-high-performance liquid chromatography on an ACQUITY UPLC BEH C18 analytical column (Waters, Elstree, UK) following mixed mode solid phase extraction. Negative ions were monitored on a QTRAP 5500 (SCIEX, Macclesfield, UK) in multiple reaction monitoring mode.

### Endpoint analysis

Changes in BoE only were expected at day 90, therefore NNAL excretion was pre-specified as the primary endpoint for between-group statistical comparisons at day 90, with the remaining BoE assigned as secondary endpoints. This inferential statistical analysis was to be repeated at day 180 for any BoE endpoint which did not reach significance at day 90. 8-Epi-PGF2α type III was pre-specified as the primary BoPH endpoint at day 180, with 11-dTx B2, FeNO and WBC also included in the inferential statistical analysis as secondary endpoints. Whilst also assigned as secondary study endpoints, sICAM-1, HDL and FEV_1_ were not planned for inclusion in the formal statistical analysis.

### Statistical methods

A full statistical analysis plan including power calculation methods has been published previously [21]. Based on the power calculation, 466 smokers in total were enrolled, with the objective of having a minimum of 50 participants complete the study in full (i.e., through to day 360, with no major protocol deviations) in each of Groups A, B (CEVal-compliant) and D. 40 never-smokers were also enrolled with the aim of 30 such participants completing the study, since this was considered sufficient to characterise biomarker levels in a never-smoker population.

Analyses were conducted on the per-protocol (PP) and CEVal-compliant populations; for details of participant composition in data tables refer to Supplementary Table 1. In summary, BoE and BoPH levels were computed at each timepoint, and changes from baseline at day 90 and/or day 180 between the THP switching group (Group B) and the continued smoking group (Group A) compared using specific contrast tests from statistical models adjusted for baseline measurements. Data are presented separately for the CEVal-compliant (indicated by CEVal levels in Group B < 78 pmol/g Hb at day 90 and < 54 pmol/g Hb at day 180) and the per-protocol (i.e., all participants who had a valid assessment of a biomarker variable and completed the study to the relevant timepoint without major protocol deviations) populations.

Alpha level across timepoints was adjusted using the O’Brien-Fleming approach [34] with overall value set at 0.0006 and 0.0151 for days 90 and 180, respectively. Any primary endpoint yielding a significant outcome at any timepoint was not to be statistically assessed at subsequent timepoints and its alpha level would be equally divided among the remaining primary endpoints. NNAL was significant at day 90; its day 180 alpha level was therefore distributed between the other primary endpoints, and as one primary endpoint (AIx) was removed from the study, a conservative approach was taken, leaving α = 0.00755 at day 180. Multiplicity adjustment for family-wise error was performed using Holm’s method [35].

Data for some of the BoE and BoPH endpoints were better represented by a log-normal distribution than a normal distribution. Therefore, after back transformation to the original scale, ratios of geometric mean partial least squares and confidence intervals were calculated. For NNN, several extreme values were present and an ancillary analysis was performed using a non-parametric (Kruskal–Wallis) test, to avoid distributional assumptions.

Missing values were not imputed and values below the analytical limit of detection or lower limit of quantification were replaced with half of the threshold values. Data analysis was performed using SAS^®^ Version 9.4.

## Results

### Participant demographics

The first participant was enrolled onto the study on 7th March 2018, and recruitment was completed on 31st March 2019. Of smokers with no intent to quit, 79 were randomised to Group A and 197 to Group B, and 190 smokers intending to quit were enrolled into Group D. Of these, 20 in Group A, 70 in Group B, and 81 in Group D were withdrawn before or missed their day 180 visit. Thus, 59, 127, and 109, respectively, were included in the day 180 analysis. 40 never-smokers were enrolled into Group E; 3 of these participants withdrew from the study prior to the Day 180 visit and as such 37 are included in the day 180 analysis.

Brief demographic details for participants in all groups are presented in Supplementary Table 2. The average age of participants in each group ranged from 37 to 40 years of age, and the overall male:female gender split was 180:152 with only minor differences between groups. Self-reported baseline cigarette consumption was broadly similar across Groups A, B and D, as was total FTCD score. Participants were predominantly white (86.4–89.9% depending on study group), and there were no notable differences in age, weight or BMI between study groups.

### Cigarette and neostick consumption

In Group A, self-reported cigarette consumption at all timepoints up to day 180 remained largely similar to that reported at screening (Table 1). In Group B, consumption of Neosticks was slightly higher than usual brand combustible cigarette consumption reported at screening and in Group A at all timepoints but remained stable over time to day 180 (Table 1). In Groups B and D, self-reported cigarette consumption was very low following either switching to the THP (Group B) or being required to abstain from all nicotine/tobacco product use (Group D).

**Table 1:** Consumption data for study participants in the day 180 per-protocol population

Numbers	Group A (continue to smoke)			Group B (switch to THP^a^)			Group D (cessation)		
	Baseline	Day 90	Day 180	Baseline	Day 90	Day 180	Baseline	Day 90	Day 180
CC consumption^b^									
Number of participants	59	59	59	127	122^d^	122^d^	109	100^d^	104^d^
Mean (SD)	18.0 ± 5.2	17.3 ± 5.3	17.4 ± 4.6	17.9 ± 5.1	0.2 ± 1.4	0.0 ± 0.1	18.1 ± 5.4	0.1 ± 0.7	0.0 ± 0.1
Minimum	10.0	7.4	8.7	10.0	0.0	0.0	10.0	0.0	0.0
Maximum	30.0	30.0	27.8	30.0	15.5	0.7	30.0	6.7	0.7
Neostick consumption^c^									
Number of participants	–	–	–	–	123^e^	127	–	–	–
Mean (SD)	–	–	–	–	20.8 ± 9.0	21.9 ± 9.7	–	–	–
Minimum	–	–	–	–	0.7	0.4	–	–	–
Maximum	–	–	–	–	53.5	53.8	–	–	–

Data were recorded at ± 3 days up to day 90 or ± 14 days after day 90 due to individual participant visit scheduling. For cigarette consumption, data were averaged using daily self-reported consumption across all days between the relevant study clinic visits. Baseline combustible cigarette consumption data were self-reported by participants at screening. For THP consumption, the number of Neosticks dispensed at a participant visit minus the number of sticks returned at the subsequent visit was divided by the number of days between the two visits

^a^*THP* tobacco heating product

^b^Average number of conventional cigarettes (CC) smoked per day

^c^Average number of neosticks used per day

^d^Some participants failed to self-report consumption data (see Supplementary Table 1)

^e^Consumption data for four participants could not be calculated at Day 90 (see Supplementary Table 1). For details of participant composition refer to Supplementary Table 1

### Compliance

CEVal measurement indicated compliance in 97 (76%) of the 127 participants in Group B reaching Day 180. Although only used, as planned, to specify a compliant subset of Group B, CEVal levels in Group D participants would indicate compliance in 80 (73%) of the 109 participants in this group reaching Day 180. At baseline, only three never-smokers had CEVal concentrations above the assay LLOQ of 2 pmol/g globin; their concentrations were 2.4, 2.5 and 5.5 pmol/g globin. At Day 90, all but two never-smokers (3.5 and 4.6 pmol/g globin) had CEVal concentrations below the assay LLOQ. At Day 180, only four never-smokers had CEVal concentrations at or above the assay LLOQ (4.6, 2.6, 2.0 and 2.0 pmol/g globin).

### Biomarkers of exposure and of potential harm

**Fig. 1:**
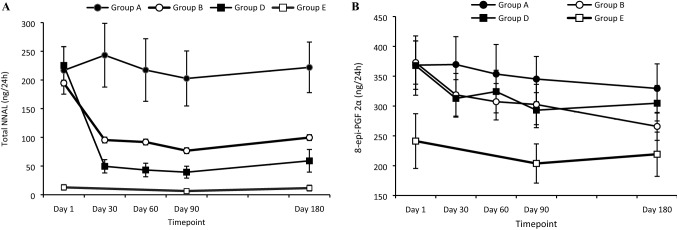
Time-series plots of changes in primary endpoints in the day 180 per-protocol population. Data are means ± 95% confidence intervals for the BoE Total 4-(methylnitrosamino)-1-(3-pyridyl)-1-butanol (NNAL; panel **A**) and the BoPH 8-epi-Prostaglandin F2a type III (8-Epi-PGF2α type III; Panel **B**). Data shown are for the Day 180 PP population (CEVal-compliant for Group B), excluding any invalid data points (e.g., urine collection issues, prohibited concomitant medication) and, for panel **B**, two extreme outliers (2543 and 2190 ng/24 h) for Group E. Therefore *N* = 52–56 (Group A), 84–90 (Group B), 97–107 (Group D), 31–36 (Group E). Group A, continue to smoke combustible cigarettes; Group B, switch to THP; Group D, cessation; Group E, never smokers

Time-series data for the two BoE and BoPH assessed as primary endpoints (NNAL and 8-Epi-PGF2α type III) among CEVal-compliant participants in Group B and among the per-protocol population in Groups A, D and E are presented in Fig. 1. Levels of NNAL (Fig. 1A) in Group A remained similar to baseline over time. In contrast, levels were reduced by approximately 50% in Group B (switch to THP) and approximately 80% in Group D (cessation) by day 30, with these exposure reductions maintained at similar levels between days 30 and 180. For 8-Epi-PGF2α type III, levels trended towards a slight reduction between baseline and day 180 in Group A, whereas in Groups B and D levels reduced gradually to a greater extent over time, with a total drop of approximately 29% and 17% by day 180, respectively (Fig. 1B). In Group E, both NNAL and 8-Epi-PGF2α type III levels remained constant over time (Fig. 1).

Statistical analyses of the differences in BoE and BoPH changes from baseline between groups A and B in the CEVal-compliant PP population are presented in Table 2; the BoE analyses were only performed on day 180 data if differences at day 90 were not significant, as per the SAP. A complete listing of mean BoE levels in Group A and CEVal-compliant Group B at baseline, day 90 and day 180 is presented in Supplementary Table 3. For those BoPH for which inferential statistical analyses were not performed (sICAM-1, HDL and FEV_1_) descriptive statistics are presented in Table 3. Reductions in the levels of all BoE were seen in Group B between baseline and day 180. When compared to changes in Group A, the reductions in the THP group were statistically significant at day 90 for NNAL, 3-HPMA, HMPMA, MHBMA, HEMA, 4-ABP, 2-AN, *o-*Tol, 1-OHP, eCO, S-PMA and CEMA (Table 2). Changes from baseline for NNN or TNeq were not statistically significant between Groups A and B at days 90 or 180 following multiple comparisons adjustment (Table 2). In the case of NNN, this was despite reductions in exposure from baseline in Group B of 57% at day 90 and 38% at day 180 (Table 2), compared with reductions in Group A of 16% at day 90 and 3% at day 180. One potential reason for the lack of statistical significance for NNN levels between Groups A and B is that NNN levels in Group B were skewed due to an extreme observation at day 180 (456.26 ng/24 h) relative to the mean group B value of 11.34 ng/24 h. A Kruskal–Wallis test suggested reductions in exposure to NNN in Group B (*p* = 0.0103) compared to continued smoking, and this reduction was enhanced (*p *= 0.0061) after removal of the most extreme value in Group B, in the absence of multiple comparison adjustments.

**Table 2:** Between-group statistical analysis of change from baseline in BoE and BoPH in the CEVal-compliant per-protocol population

Biomarker (units)	Group^a^	*N*^b^	Day	LS^c^ mean or GLS^d^ mean ratio compared to baseline^e^	Difference between groups (CI)^e,f^	*p* value^g^
Total NNAL^e,h^ (ng/24 h)	A	55	90	0.89	0.50 (0.33, 0.75)	< 0.0001
	B	97		0.44		
Total NNN^e,^^i^ (ng/24 h)	A	55	90	0.84	0.51 (0.24, 1.10)	0.0025
	B	97		0.43		
Total NNN^e,^^i^ (ng/24 h)	A	53	180	0.97	0.64 (0.37, 1.10)	0.0276
	B	85		0.62		
3-HPMA^e,^^j^ (μg/24 h)	A	55	90	1.00	0.30 (0.19, 0.48)	< 0.0001
	B	97		0.30		
HMPMA^e,^^k^ (μg/24 h)	A	55	90	0.85	0.29 (0.19, 0.44)	< 0.0001
	B	97		0.25		
MHBMA^e,l^ (μg/24 h)	A	55	90	1.05	0.11 (0.05, 0.23)	< 0.0001
	B	97		0.11		
HEMA^e,m^ (μg/24 h)	A	55	90	0.83	0.52 (0.30, 0.88)	< 0.0001
	B	97		0.43		
4-aminobiphenyl (ng/24 h)^e^	A	55	90	0.86	0.29 (0.19, 0.42)	< 0.0001
	B	97		0.25		
2-aminonaphthalene (ng/24 h)^e^	A	55	90	0.88	0.15 (0.09, 0.25)	< 0.0001
	B	97		0.13		
*o*-Toluidine (ng/24 h)^e^	A	55	90	1.01	0.36 (0.24, 0.52)	< 0.0001
	B	97		0.36		
1-hydroxypyrene (ng/24 h)^e^	A	55	90	1.15	0.37 (0.24, 0.57)	< 0.0001
	B	97		0.42		
FeNO^e,n^ (ppb)	A	54	180	0.99	1.52 (1.20, 1.93)	< 0.0001
	B	93		1.51		
WBC^e,o^ count (10^9^/L)	A	56	180	0.99	0.85 (0.76, 0.94)	< 0.0001
	B	93		0.84		
eCO^p,q^ (ppm)	A	62	120/150	15.06	−13.37 (−16.20, −10.54)	< 0.0001
	B	112		1.69		
TNeq^p.r^ (mg/24 h)	A	55	90	−1.67	−3.11 (−8.74, 2.53)	0.0550
	B	97		−4.77		
TNeq^p,r^ (mg/24 h)	A	53	180	−4.70	−1.13 (−5.21, 2.95)	0.4529
	B	85		−5.84		
S-PMA^p,s^ (μg/24 h)	A	55	90	−0.74	−2.84 (−4.51, −1.18)	< 0.0001
	B	97		−3.58		
CEMA^p,t^ (μg/24 h)	A	55	90	−2	−158 (−212, −103)	< 0.0001
	B	97		−159		
11-dTx B2^p,u^ (ng/24 h)	A	53	180	−100	−173 (−399, 53)	0.0396
	B	85		−274		
8-Epi-PGF2α^p,v^ (ng/24 h)	A	53	180	−41	−76 (−144, −7)	0.0032
	B	85		−116		

All analyses, except for eCO, were performed using biomarker levels at baseline (day 1) and on either day 90 or day 180, as indicated. eCO was analysed as the difference between the means of absolute values on days 120 and 150

^a^Group A, continue to smoke combustible cigarettes, Group B, switch to THP

^b^*N*, number of participants (for details of participant composition refer to Supplementary Table 1)

^c^*LS* least squares

^d^*GLS* geometric least squares

^e^GLS mean and ratio shown for data log-transformed prior to calculation of change from baseline

^f^*CI* confidence interval: 99.94% CI shown for day 90; 99.245% CI shown for day 180

^g^Significance threshold 0.0006 on day 90 and 0.00755 on day 180

^h^*NNAL* 4-(methylnitrosamino)-1-(3-pyridyl)-1-butanol

^i^*NNN* *N*-nitrosonornicotine

^j^*3-HPMA* 3-hydroxypropylmercapturic acid

^k^*HMPMA* 3-hydroxy-1-methylpropylmercapturic acid

^l^*MHBMA* monohydroxybutenyl-mercapturic acid

^m^*HEMA* 2-hydroxyethylmercapturic acid

^n^*FeNO* fractional exhaled nitric oxide

^o^*WBC* white blood cell

^p^LS mean and difference shown for untransformed data

^q^*eCO* exhaled carbon monoxide

^r^*TNeq* total nicotine equivalents (nicotine, cotinine, 3-hydroxycotinine and their glucuronide conjugates)

^s^*S-PMA* S-phenylmercapturic acid

^t^*CEMA* 2-cyanoethylmercapturic acid

^u^*11-dTx B2* 11-dehydrothromboxane B2

^v^*8-Epi-PGF2α* 8-epi-prostaglandin F2α type III

**Table 3:** Descriptive Statistics for sICAM-1, HDL and FEV1 at Baseline (day 1), Day 90 and Day 180 in the Day 180 CEVal-compliant per-protocol population

Biomarker (units)	Group^a^	*N*^b^	Day 1	Day 90	Day 180
sICAM-1^c^ (ng/mL)	A	58–59	474.3 (444.4, 504.2)	491.0 (454.9, 527.1)	501.8 (463.6, 540.0)
	B	94–97	464.4 (437.6, 491.1)	428.0 (409.1, 447.0)	433.2 (410.3, 456.1)
HDL^d^ (mmol/L)	A	58–59	1.39 (1.29, 1.49)	1.39 (1.28, 1.49)	1.37 (1.26, 1.49)
	B	94–97	1.41 (1.34, 1.48)	1.49 (1.41, 1.57)	1.48 (1.40, 1.56)
FEV_1_%pred^e^	A	55–58	91.5 (88.5, 94.5)	90.1 (87.0, 93.1)	88.1 (85.1, 91.0)
	B	89–93	91.9 (89.7, 94.2)	92.8 (90.5, 95.1)	93.0 (90.8, 95.1)

Data are means with lower and upper 95% confidence intervals in parentheses

^a^Group A continue to smoke combustible cigarettes, Group B, switch to THP

^b^*N*, number of participants (for details of participant composition refer to Supplementary Table 1)

^c^*sICAM-1* soluble intercellular adhesion molecule-1

^d^*HDL* high-density lipoprotein

^e^*FEV*_*1*_*%pred* forced expiratory volume in 1 s percentage of predicted

Regarding BoPH in the Group B CEVal-compliant PP population, 8-Epi-PGF2α type III levels and WBC count were reduced, and FeNO was elevated, between baseline and day 180. These effects were significant when comparing Groups A and B (Table 2). Levels of 11-dTX B2 were lower at day 180 than at baseline in Group B. Despite this reduction being over two-and-a-half times that seen in Group A, the comparison with Group A did not reach statistical significance (Table 2).

For other BoPH, for which only descriptive statistics were generated, favourable directional trends were seen over time in participants who switched to using the THP (Group B; Table 3). Thus, compared to baseline sICAM-1 was lower on days 90 and day 180 while both HDL and FEV_1_ were increased. This contrasted with elevation of sICAM-1 and reductions in HDL and FEV_1_ over time in continued smokers (Group A).

Complete listings of mean BoE levels in Groups A, B, D and E at baseline, day 90 and day 180, and statistical analyses of BoE and BoPH data, in the total PP population are presented in Supplementary Tables 4 and 5. There were no major differences in statistical outcomes between the total PP and CEVal-compliant PP populations. Thus, significant differences were seen at day 90 between Groups A and B for reductions in the BoE NNAL, 3-HPMA, HMPMA, MHBMA, HEMA, 4-ABP, 2-AN, *o*-Tol, 1-OHP, eCO, S-PMA and CEMA, and for the changes in the BoPH 8-Epi-PGF2α type III, FeNO and WBC count at day 180. As seen in the CEVal-compliant PP population, the reduction in 11-dTx B2 neared statistical significance in the PP population. Finally, there were no major differences in the descriptive statistics for the BoPH sICAM-1, HDL and FEV_1_ between the PP and CEVal-compliant PP populations (Supplementary Table 6).

### Adverse events

Up to Day 180, exposure period adverse events occurred in 329 participants, including 5 serious adverse events considered unrelated to any study product. The most frequently reported adverse event was headache, and the majority of adverse events were mild or moderate in severity.

## Discussion

In a previous publication of a planned, interim analysis of a subset of study participants at day 90 from this study, and also in a publication assessing data from a 5-day confinement study, we demonstrated significant reductions in exposure to a number of cigarette smoke toxicants in smokers switching to using the THP [12, 13]. These exposure reductions were correlated with the lower THP emissions compared to cigarette smoke and approached those seen with smoking cessation for a number of the BoE examined. Here, we build on those observations by reporting reductions in BoE in the full population of study participants at day 90 while also demonstrating that exposure reductions persisted at day 180. The day 180 BoE reductions were to a degree similar to that in the smoking cessation group in the per-protocol population (Supplementary Table 5). Importantly, we also demonstrate that exposure reductions in those switching to using the THP were accompanied by significant changes in BoPH, which are associated with disease risk and therefore considered to indicate changes in smoking-related harm [15], compared with those who continued smoking. Furthermore, although no formal statistical analyses have been performed and descriptive statistics have been presented, it is notable that in the per-protocol population changes in BoPH at Day 180 were directionally similar in the THP switching group to those seen in the smoking cessation group.

When compared with continued smoking, significant reductions were seen between baseline and day 180 for 8-Epi-PGF2α type III (a prostaglandin associated with systemic oxidative stress and implicated in smoking-related disease progression [36‒38]) and white blood cell count (an inflammatory marker indicative of cardiovascular disease risk [39]), while FeNO (an indicator of airway inflammation, lung health and vascular tone [40]) levels were significantly increased. Furthermore, urinary NNAL levels were significantly reduced between baseline and day 180 and while this indicates a reduction in exposure to the tobacco-specific nitrosamine NNK, urinary NNAL levels are also considered a biomarker for lung cancer risk [30, 32, 33]. Of interest, although as per the SAP the statistical significance was not assessed, we observed an increase in HDL at day 90 and day 180 in the THP switching group. Given the rough proportionality of increased HDL levels with reduced CVD risk, this change could be biologically relevant [41]. Overall, taking our BoE and BoPH findings into account our data are indicative that complete switching from cigarette smoking to using a THP could reduce the risk of smoking-related diseases.

While the criteria under which a tobacco product may be considered a reduced exposure or reduced risk tobacco product have not been fully defined, the US IoM and the FDA [16, 17] have indicated one potential criterion, observation of a statistically significant difference in BoE and BoPH in those switching to using a novel product compared with continued smoking, and similarity of effect size compared with cessation may also be considered a criterion [16]. Indeed, the FDA recently authorized the marketing of a THP as a MRTP with ‘Reduced Exposure information’ based in part on these criteria [42]. Many of the BoE examined in this study meet either or both of these criteria, as do the BoPH 8-Epi-PGF2α type III, WBC count, FeNO, NNAL, 11-dTx B2, sICAM-1, HDL and FEV_1_ which all changed favourably in the THP switching group. Furthermore, the BoPH changes meet a criterion of biological relevance suggested by Chang et al. [15] such that differences > 10% can distinguish between smokers and non-smokers. Our findings add to the body of evidence suggesting that THPs are potentially MRTPs when compared to combustible cigarette smoking. Our findings may also provide insight into the utility of certain biomarkers for assessing changes in smoking-related disease risk. For example, one of the BoPH assessed in this study, 8-Epi-PGF2α type III, showed a large degree of variability between timepoints, even within the never-smoker group (Fig. 1). This has also been observed previously in other switching studies [23]. While the reasons for such variability cannot be ascertained, potentially this could be due to 8-Epi-PGF2α type III being a general marker of systemic oxidative stress, and therefore being susceptible to change due to factors other than changes in cigarette smoking status (e.g., other risk factors or seasonal disorders). While such variability may hinder data analysis and interpretation, it does give insight into how future studies should be designed to take into account such variability, for example by ensuring an adequate sample size.

While a previous study reported BoPH changes in smokers switching to using a THP [27], self-reported compliance was as low as 50%. Furthermore, cigarettes could have constituted up to 30% of tobacco products used in participants defined as complete THP switchers. These issues lessened the ability to detect changes in BoPH. A strength of this current study is the use of a biochemical measure (CEVal) and pre-determined thresholds to determine compliance [21], allowing us to define a group of complete switchers in which to assess biomarker changes. Additionally, in this study, we were able to maintain compliance at higher levels using both participant selection (high intentions to quit smoking in the abstinence group) and participant monitoring. It is notable in this regard that potentially due to this maintenance of compliance there were no major differences in our findings between the CEVal-compliant and the per-protocol analysis populations.

While the degree of compliance and its accurate assessment are strengths of our study, there are some limitations of the study and our findings. While we provide evidence of both acute [12] and sustained reductions in BoE and BoPH in smokers switching to THP use, the findings do not necessarily indicate changes in population-level exposure or risk, particularly if within those populations smokers do not switch completely and instead switch to dual-using cigarettes and THPs. Secondly, the generalizability of our findings may be limited since the study involved a young, healthy population and a small sample size. Larger, future studies in other populations are needed to improve the generalisability and strengthen our conclusions regarding reduced disease risk. Furthermore, while BoE for a number of smoke toxicants linked with smoking-related disease were reduced, and these were associated with favourable changes in BoPH covering a spectrum of smoking-related diseases, only limited conclusions can be drawn regarding whether switching to THP use reduces smoking-related morbidity and mortality. Such information can only come from prospective longer-term epidemiological and/or cohort studies.

In summary, the data presented here build on our prior work by demonstrating that exposure changes in smokers switching to using the THP were sustained and extend this finding by demonstrating that these exposure reductions were associated with beneficial changes in disease risk biomarkers covering several smoking-related diseases. While the use of THPs is likely not risk-free and may be addictive due to nicotine delivery to users, and given that to eliminate the risks associated with cigarette smoking the best course of action for a smoker to take is to completely abstain from the use of any tobacco products [1], our study gives an insight into the potential beneficial effects of smoking-related disease reduction in smokers switching to using THPs. This is illustrated by the similarity in BoE in smokers who switched to using the THP or who quit all tobacco/nicotine use. When taking into account established criteria for risk reduction, our data add support to the body of evidence suggesting that THPs are potential MRTPs and also support the notion that the deleterious health impacts of cigarette smoking may be reduced in smokers who completely switch to using THPs. Further research, including assessments of disease endpoints such as cardiac or respiratory events in smokers who switch to using THPs, may be able to further determine this risk reduction potential.

## Supplementary Information

Below is the link to the electronic supplementary material.

Supplementary file1 (PDF 607 KB)

## Data Availability

Deidentified participant-level data will be available on request in SDTM format. This data will be available immediately following publication for at least 5 years. Data will be available to anyone who wishes access to the data and for any purpose. Requests for data should be made to clinical_info@bat.com and data requestors must sign a data access agreement.
