# Supplementary material for: Changes in biomarkers after 180 days of tobacco heating product use: a randomised trial
Source: Intern Emerg Med. 2021 Jul 1;16(8):2201–12. doi: 10.1007/s11739-021-02798-6 (PMC8563516; doi:10.1007/s11739-021-02798-6)
Supplement: Supplementary file 1 — Supplementary file1 (PDF 607 KB) [file 11739_2021_2798_MOESM1_ESM.pdf]

**Supplementary Table 1. Rationale for Participant Composition and Sample Sizes in Data Tables**

| Table | Endpoint | Group | Participant selection/sample size |
|---|---|---|---|
| 1 | **Cigarette consumption** | A | Consists of the full Day 180 PP population, n = 59. |
| | **Cigarette consumption** | B | Of the 127 participants in the Day 180 PP population, 5 participants at the Day 90 timepoint and 5 participants at the Day 180 timepoint failed to self-report their cigarette consumption on any of the preceding 30 days, thus n = 122 at both Day 90 and Day 180. |
| | **Neostik consumption** | | Of the 127 participants in the Day 180 PP population, 4 participants could not have consumption data calculated at Day 90 due to missed visits, therefore n = 123 at Day 90. |
| | **Cigarette consumption** | D | Of the 109 participants in the Day 180 PP population, 9 participants at the Day 90 timepoint and 5 participants at the Day 180 timepoint failed to self-report their cigarette consumption on any of the preceding 30 days, thus n = 100 and n = 104, respectively. |
| 2 | **Urinary markers at Day 90 (NNAL, NNN, 3-HPMA, HMPMA, MHBMA, HEMA, 4-ABP, 2-AN, o-Tol, 1-OHP, TNeq, S-PMA, CEMA)** | A | Of the 63 participants in the Day 90 PP population, 8 were excluded from the analysis (1 x prohibited medication affecting baseline and/or Day 90 visit, 7 x urine collection issues affecting baseline and/or Day 90 visit) thus the analysis was performed on n = 55 participants |
| | | B | Of the 141 participants in the Day 90 PP population, 27 were excluded from the analysis (3 x prohibited medication affecting baseline and/or Day 90 visit, 21 x urine collection issues affecting baseline and/or Day 90 visit, 3 x baseline and/or Day 90 samples not analysed in error). Of the remaining participants, 17 were |

| | | | |
|---|---|---|---|
| | | | not CEVal-compliant at Day 90, thus the analysis was performed on n = 97 participants. |
| **2** | **Urinary markers at Day 180 (NNN, TNeq, 11-dTx B2, 8-Epi-PGF2α)** | A | Of the 59 participants in the Day 180 PP population, 6 were excluded from the analysis (urine collection issues affecting baseline and/or Day 180 visit) thus the analysis was performed on n = 53 participants. |
| | | B | Of the 127 participants in the Day 180 PP population, 21 were excluded from the analysis (1 x prohibited medication affecting baseline and/or Day 180 visit, 20 x urine collection issues affecting baseline and/or Day 180 visit). Of the remaining participants, 21 were not CEVal-compliant at Day 180, thus the analysis was performed on n = 85 participants. |
| **2** | **WBC at Day 180** | A | Of the 59 participants in the Day 180 PP population, 3 were excluded from the analysis (baseline and/or Day 180 samples not received at lab) thus the analysis was performed on n = 56 participants. |
| | | B | Of the 127 participants in the Day 180 PP population, 7 were excluded from the analysis (1 x prohibited medication affecting baseline and/or Day 180 visit, 6 x baseline and/or Day 180 samples not received at lab). Of the remaining participants, 27 were not CEVal-compliant at Day 180, thus the analysis was performed on n = 93 participants. |
| **2** | **FeNO at Day 180** | A | Of the 59 participants in the Day 180 PP population, 5 were excluded from the analysis (baseline and/or Day 180 assessment not performed by site) thus the analysis was performed on n = 54 participants. |
| | | B | Of the 127 participants in the Day 180 PP population, 6 were excluded from the analysis (1 x prohibited medication affecting baseline and/or Day 180 visit, 5 x baseline and/or Day 180 assessment not performed). Of the remaining participants, 28 were not CEVal-compliant at Day 180, thus the analysis was performed on n = 93 participants. |

| | | | |
|---|---|---|---|
| **2** | **eCO at Day 120/Day 150** | A | Of the 63 participants in the Day 90 PP population, 1 was excluded from the analysis since they were withdrawn prior to their Day 120 visit, thus the analysis was performed on n = 62 participants. |
| | | B | Of the 141 participants in the Day 90 PP population, 6 were excluded from the analysis (1 x prohibited medication affecting Day 120 and Day 150 visit, 5 x participants withdrew prior to Day 120 visit). Of the remaining participants, 23 were not CEVal-compliant at Day 90, thus the analysis was performed on n = 112 participants. |
| **3** | **sICAM-1 and HDL** | A | Day 1 and Day 180 consist of the Day 180 PP population (n = 59). Day 90 n = 58 consists of the Day 180 PP population excluding one participant who took a prohibited medication affecting the Day 90 visit. |
| | | B | Day 1 n = 97 consists of the Day 180 PP population, excluding 30 participants who were not CEVal compliant at Day 180. Day 90 n = 94 consists of the Day 180 PP population, excluding 3 participants who missed the Day 90 visit, 1 participant who took a prohibited medication affecting the Day 90 visit, and 29 participants who were not CEVal compliant at Day 180. Day 180 n = 96 consists of the Day 180 PP population, excluding 1 participant who took a prohibited medication affecting the Day 180 visit, and 30 participants who were not CEVal compliant at Day 180. |
| **3** | **$FEV_1$%pred** | A | Day 1 n = 58 consists of the Day 180 PP population excluding 1 participant who did not have a Day 1 result. Day 90 n = 56 consists of the Day 180 PP population excluding 3 participants who did not have a Day 90 result. Day 180 n = 55 consists of the Day 180 PP population excluding 4 participants who did not have a Day 180 result. |
| | | B | Day 1 n = 89 consists of the Day 180 PP population, excluding 10 participants who did not have a Day 1 result and 28 participants who were not CEVal compliant at Day 180. Day 90 n = 89 consists of the Day 180 PP population, excluding 10 participants who did not have a Day 90 result and 28 participants who were not CEVal compliant at Day 180. Day 180 n = 93 consists of the Day 180 PP population, excluding 5 participants who did not have a Day 180 result and 29 participants who were not CEVal compliant at Day 180. |

| | | | |
|---|---|---|---|
| **S3** | **Urinary markers at Day 1 (NNAL, NNN, 3-HPMA, HMPMA, MHBMA, HEMA, 4-ABP, 2-AN, o-Tol, 1-OHP, TNeq, S-PMA, CEMA)** | A | Of the 59 participants in the Day 180 PP population, 5 participants are excluded due to urine collection issues affecting the Day 1 visit, thus n = 54 participants. |
| | | B | Of the 127 participants in the Day 180 PP population, 14 participants are excluded due to urine collection issues affecting the Day 1 visit, and a further 23 participants were not CEVal compliant, thus n = 90 participants. |
| **S3** | **Urinary markers at Day 90 (NNAL, NNN, 3-HPMA, HMPMA, MHBMA, HEMA, 4-ABP, 2-AN, o-Tol, 1-OHP, TNeq, S-PMA, CEMA)** | A | Of the 59 participants in the Day 180 PP population, 4 participants are excluded (1 x prohibited medication affecting the Day 90 visit, 3 x urine collection issues affecting the Day 90 visit), thus n = 55 participants. |
| | | B | Of the 127 participants in the Day 180 PP population, 15 participants are excluded (3 x missed Day 90 visit, 1 x Day 90 sample not analysed in error, 1 x prohibited medication affecting the Day 90 visit, 10 x urine collection issues affecting the Day 90 visit), and a further 24 participants were not CEVal compliant, thus n = 88 participants. |
| **S3** | **Urinary markers at Day 180 (NNAL, NNN, 3-HPMA, HMPMA, MHBMA, HEMA, 4-ABP, 2-AN, o-Tol, 1-OHP, TNeq, S-PMA, CEMA)** | A | Of the 59 participants in the Day 180 PP population, 3 participants are excluded (1 x no urine collected at Day 180 visit, 2 x urine collection issues affecting the Day 180 visit), thus n = 56 participants. |
| | | B | Of the 127 participants in the Day 180 PP population, 10 participants are excluded (1 x no urine collected at Day 180 visit, 1 x prohibited medication affecting the Day 180 visit, 8 x urine collection issues affecting the Day 180 visit), and a further 27 participants were not CEVal compliant, thus n = 90 participants. |
| **S3** | **eCO at Day 1** | A | Consists of the full Day 180 PP population, n = 59. |
| | | B | Of the 127 participants in the Day 180 PP population, 30 participants were not CEVal compliant, thus n = 97 participants. |
| **S3** | **eCO at Day 90** | A | Of the 59 participants in the Day 180 PP population, one participant is excluded due to a prohibited medication affecting their Day 90 visit, therefore n = 58. |
| | | B | Of the 127 participants in the Day 180 PP population, 4 participants are excluded (3 x missed Day 90 visit, 1 x prohibited medication affecting the Day 90 visit), and a further 26 participants were not CEVal compliant, thus n = 97 participants. |
| **S3** | **eCO at Day 180** | A | Consists of the full Day 180 PP population, n = 59. |

| | | | |
|---|---|---|---|
| | | B | Of the 127 participants in the Day 180 PP population, one participant is excluded due to a prohibited medication affecting their Day 180 visit, and a further 30 subjects were not CEVal compliant, therefore n = 96. |
| **S4** | **Urinary markers at Day 1 (NNAL, NNN, 3-HPMA, HMPMA, MHBMA, HEMA, 4-ABP, 2-AN, o-Tol, 1-OHP, TNeq, S-PMA, CEMA)** | A | Of the 63 participants in the Day 90 PP population, 6 participants are excluded due to urine collection issues affecting the Day 1 visit, thus n = 57. |
| | | B | Of the 141 participants in the Day 90 PP population, 18 participants are excluded (1 x prohibited medication affecting the Day 1 visit, 16 x urine collection issues affecting the Day 1 visit, 1 x Day 1 sample not analysed in error), thus n = 123. |
| | | D | Of the 136 participants in the Day 90 PP population, 19 participants are excluded (18 x urine collection issues affecting the Day 1 visit, 1 x Day 1 sample not analysed in error), thus n = 117. |
| | | E | Of the 37 participants in the Day 90 PP population, 4 participants are excluded due to urine collection issues affecting the Day 1 visit, thus n = 33. |
| **S4** | **Urinary markers at Day 90 (NNAL, NNN, 3-HPMA, HMPMA, MHBMA, HEMA, 4-ABP, 2-AN, o-Tol, 1-OHP, TNeq, S-PMA, CEMA)** | A | Of the 63 participants in the Day 90 PP population, 4 participants are excluded (1 x prohibited medication affecting the Day 90 visit, 3 x urine collection issues affecting the Day 90 visit), thus n = 59 participants. |
| | | B | Of the 141 participants in the Day 90 PP population, 15 participants are excluded (2 x prohibited medication affecting the Day 90 visit, 10 x urine collection issues affecting the Day 90 visit, 3 x Day 90 samples not analysed in error), thus n = 126 participants. |
| | | D | Of the 136 participants in the Day 90 PP population, 12 participants are excluded (6 x urine collection issues affecting the Day 90 visit, 1 x prohibited medication affecting the Day 90 visit, 5 x Day 90 samples not analysed in error), thus n = 124. |
| | | E | Of the 37 participants in the Day 90 PP population, 1 participant is excluded due to a urine collection issue affecting the Day 90 visit, thus n = 36. |
| **S4** | **Urinary markers at Day 180 (NNAL, NNN, 3-HPMA, HMPMA, MHBMA, HEMA, 4-ABP, 2-AN, o-Tol, 1-OHP, TNeq, S-PMA, CEMA)** | A | Of the 59 participants in the Day 180 PP population, 3 participants are excluded (1 x no urine collected at Day 180 visit, 2 x urine collection issues affecting the Day 180 visit), thus n = 56 participants. |
| | | B | Of the 127 participants in the Day 180 PP population, 10 participants are excluded (1 x no urine collected at Day 180 visit, 1 x prohibited medication affecting the Day 180 visit, 8 x urine collection issues affecting the Day 180 visit), thus n = 117 participants. |

| | | | |
|---|---|---|---|
| | | D | Of the 109 participants in the Day 180 PP population, 2 participants are excluded (1 x urine collection issue affecting Day 180 visit, 1 x prohibited medication affecting the Day 180 visit), thus n = 107. |
| | | E | Of the 37 participants in the Day 180 PP population, 4 participants are excluded due to urine collection issues affecting the Day 180 visit, thus n = 33. |
| **S4** | **eCO at Day 1** | A | Consists of the full Day 90 PP population, n = 63. |
| | | B | Of the 141 participants in the Day 90 PP population, 1 participant is excluded due to a prohibited medication affecting the Day 1 visit, thus n = 140. |
| | | D | Consists of the full Day 90 PP population, n = 136. |
| | | E | Consists of the full Day 90 PP population, n = 37. |
| **S4** | **eCO at Day 90** | A | Of the 63 participants in the Day 90 PP population, one participant is excluded due to a prohibited medication affecting their Day 90 visit, therefore n = 62. |
| | | B | Of the 141 participants in the Day 90 PP population, 2 participants are excluded due to a prohibited medication affecting their Day 90 visit, therefore n = 139. |
| | | D | Of the 136 participants in the Day 90 PP population, 2 participants are excluded (1 x prohibited medication affecting the Day 90 visit, 1 x assessment not performed), therefore n = 134. |
| | | E | Consists of the full Day 90 PP population, n = 37. |
| **S4** | **eCO at Day 180** | A | Consists of the full Day 180 PP population, n = 59. |
| | | B | Of the 127 participants in the Day 180 PP population, one participant is excluded due to a prohibited medication affecting their Day 180 visit, therefore n = 126. |
| | | D | Of the 109 participants in the Day 180 PP population, one participant is excluded due to a prohibited medication affecting their Day 180 visit, therefore n = 108. |
| | | E | Consists of the full Day 180 PP population, n = 37. |
| **S5** | **All** | A | As per Table 2. |

| | | | |
|---|---|---|---|
| | | B | As per Table 2 except participants who were not CEVal-compliant were not removed from the analyses. |
| **S5** | **Urinary markers at Day 90 (NNAL, NNN, 3-HPMA, HMPMA, MHBMA, HEMA, 4-ABP, 2-AN, o-Tol, 1-OHP, TNeq, S-PMA, CEMA)** | D | Of the 136 participants in the Day 90 PP population, 28 were excluded from the analysis (22 x urine collection issues affecting baseline and/or Day 90 visit, 1 x prohibited medication affecting the Day 90 visit, 5 x Day 90 samples not analysed due to other non-compliance affecting the Day 90 visit) thus the analysis was performed on n = 108 participants. |
| **S5** | **Urinary markers at Day 180 (NNN, TNeq, 11-dTx B2, 8-Epi-PGF2$\alpha$)** | D | Of the 109 participants in the Day 180 PP population, 13 were excluded from the analysis (12 x urine collection issues affecting baseline and/or Day 180 visit, 1 x prohibited medication affecting the Day 180 visit) thus the analysis was performed on n = 96 participants. |
| **S5** | **WBC at Day 180** | D | Of the 109 participants in the Day 180 PP population, 9 were excluded from the analysis (1 x prohibited medication affecting the Day 180 visit, 8 x baseline and/or Day 180 samples not received at lab) thus the analysis was performed on n = 100 participants. |
| **S5** | **FeNO at Day 180** | D | Of the 109 participants in the Day 180 PP population, 10 were excluded from the analysis (1 x prohibited medication affecting the Day 180 visit, 9 x Day 180 assessment not performed) thus the analysis was performed on n = 99 participants. |
| **S6** | **sICAM-1 and HDL** | A | Day 1 n = 63 consists of the Day 90 PP population. Day 90 n = 62 consists of the Day 90 PP population excluding one participant who took a prohibited medication affecting the Day 90 visit. Day 180 n = 59 consists of the Day 180 PP population. |
| | | B | Day 1 n = 140 consists of the Day 90 PP population, excluding 1 participant who took a prohibited medication affecting the Day 1 visit. For sICAM-1, a further participant did not have a Day 1 result thus n = 139. Day 90 n = 139 consists of the Day 90 PP population, excluding 2 participants who took a prohibited medication affecting the Day 90 visit. Day 180 n = 126 consists of the Day 180 PP population, excluding 1 participant who took a prohibited medication affecting the Day 180 visit. |
| | | D | Day 1 n = 135 consists of the Day 90 PP population, excluding 1 participant who did not have a Day 1 result. For sICAM-1, a further 5 participants did not have a |

| | | | |
|---|---|---|---|
| | | | Day 1 result thus n = 130. Day 90 n = 134 consists of the Day 90 PP population, excluding 1 participant who did not have a Day 90 result and 1 participant who took a prohibited medication affecting the Day 90 visit. For sICAM-1, 1 further participant did not have a Day 90 result thus n = 133. Day 180 n = 108 consists of the Day 180 PP population, excluding 1 participant who took a prohibited medication affecting the Day 180 visit. |
| | | E | Day 1 and Day 90 consist of the full Day 90 PP population, n = 37. Day 180 consists of the full Day 180 PP population, n = 37. |
| **S6** | **$FEV_1$%pred** | A | Day 1 n = 62 consists of the Day 90 PP population excluding 1 participant who did not have a Day 1 result. Day 90 n = 60 consists of the Day 90 PP population excluding 3 participants who did not have a Day 90 result. Day 180 n = 55 consists of the Day 180 PP population excluding 4 participants who did not have a Day 180 result. |
| | | B | Day 1 n = 131 consists of the Day 90 PP population, excluding 10 participants who did not have a Day 1 result. Day 90 n = 133 consists of the Day 90 PP population, excluding 8 participants who did not have a Day 90 result. Day 180 n = 122 consists of the Day 180 PP population, excluding 5 participants who did not have a Day 180 result. |
| | | D | Day 1 n = 132 consists of the Day 90 PP population, excluding 4 participants who did not have a Day 1 result. Day 90 n = 132 consists of the Day 90 PP population, excluding 4 participants who did not have a Day 90 result. Day 180 n = 100 consists of the Day 180 PP population, excluding 9 participants who did not have a Day 180 result. |
| | | E | Day 1 and Day 90 n = 35 consists of the Day 90 PP population, excluding 2 participants at each timepoint who did not have a result. Day 180 n = 34 consists of the Day 180 population, excluding 3 participants who did not have a Day 180 result. |

Note: CEVal, N-(2-cyanoethyl) valine; NNAL, 4-(methylnitrosamino)-1-(3-pyridyl)-1-butanol; NNN, N-nitrosonornicotine; 3-HPMA, 3-hydroxypropylmercapturic acid; HMPMA, 3-hydroxy-1-methylpropylmercapturic acid; MHBMA, monohydroxybutenyl-mercapturic acid; HEMA, 2-hydroxyethylmercapturic acid; 4-ABP, 4-aminobiphenyl; 2-AN, 2-aminonaphthalene; *o*-Tol, *o*-Toluidine; 1-OHP, 1-hydroxypyrene; FeNO, fractional exhaled nitric oxide; WBC, white blood cell; eCO, exhaled carbon monoxide; TNeq, total nicotine equivalents (nicotine, cotinine, 3-hydroxycotinine and their glucuronide conjugates); S-PMA, S-phenylmercapturic acid; CEMA, 2-cyanoethylmercapturic acid; 11-dTx B2, 11-dehydrothromboxane B2; 8-Epi-PGF2$\alpha$, 8-epi-Prostaglandin F2$\alpha$ type III; sICAM-1, soluble intercellular adhesion molecule-1; HDL, high-density lipoprotein; $FEV_1$%pred, forced expiratory volume in 1 second

percentage of predicted; PP, per protocol.  Group A, continue to smoke combustible cigarettes;  Group B, switch to THP; Group D, cessation; Group E, never-smokers.

**Supplementary Table 2. Demographic Data for Study Participants in the Day 180 Per-Protocol Population**

| | | Group | | | |
|---|---|---|---|---|---|
| | | **A (continue to smoke)** | **B (switch to THP)** | **D (cessation)** | **E (never smokers)** |
| **N (PP population)** | N | 59 | 127 | 109 | 37 |
| **Age (years)** | Mean (SD) | 37 ± 9.7 | 38 ± 8.7 | 39 ± 9.2 | 40 ± 9.9 |
| **Sex** | Male:Female | 33:26 | 69:58 | 63:46 | 15:22 |
| **Weight (males; kg)** | Mean (SD) | 81.6 ± 10.80 | 80.9 ± 11.53 | 82.2 ± 11.11 | 80.0 ± 11.64 |
| **Weight (females; kg)** | Mean (SD) | 66.7 ± 13.12 | 68.4 ± 9.56 | 65.6 ± 9.75 | 66.1 ± 8.51 |
| **BMI (kg/m$^2$)** | Mean (SD) | 25.8 ± 3.64 | 25.6 ± 3.33 | 25.4 ± 3.09 | 25.4 ± 3.04 |
| **FTCD total score** | Mean (SD) | 5 ± 1.9 | 6 ± 1.8 | 5 ± 1.8 | N/A |
| **Cigarettes per day[a]** | Mean (SD) | 18 ± 5.2 | 18 ± 5.1 | 18 ± 5.4 | N/A |
| **Race** | | | | | |
| **Asian** | N (%) | 4 (6.8%) | 8 (6.3%) | 3 (2.8%) | 2 (5.4%) |
| **Black/African American** | N (%) | 3 (5.1%) | 2 (1.6%) | 3 (2.2%) | 2 (5.4%) |
| **White** | N (%) | 51 (86.4%) | 114 (89.8%) | 98 (89.9%) | 33 (89.2%) |
| **Other** | N (%) | 1 (1.7%) | 3 (2.4%) | 5 (4.6%) | 0 (0%) |
| **Ethnicity** | | | | | |
| **Hispanic/Latino** | N (%) | 0 (0%) | 2 (1.6%) | 1 (0.9%) | 0 (0%) |
| **Not Hispanic/Latino** | N (%) | 59 (100%) | 125 (98.4%) | 108 (99.1%) | 37 (100%) |

Note: Data are as collected at screening. BMI, body mass index; N, number of participants; PP, per-protocol; SD, standard deviation; FTCD, Fagerström Test for Cigarette Dependence; N/A, not applicable. [a]self-reported cigarette consumption at screening.

**Supplementary Table 3. BoE Levels at Baseline (Day 1), Day 90 and Day 180 in the Day 180 CEVal-Compliant Per-Protocol Population**

| Biomarker (units) | Group | N | Day 1 | N | Day 90 | N | Day 180 |
|---|---|---|---|---|---|---|---|
| **Total NNAL (ng/24h)** | A | 54 | 216.72 (175.32, 258.12) | 55 | 202.63 (154.63, 250.62) | 56 | 221.94 (177.86, 266.02) |
| | B | 90 | 194.60 (164.58, 224.62) | 88 | 84.51 (66.23, 102.79) | 90 | 99.62 (85.52, 113.72) |
| **Total NNN (ng/24h)** | A | 54 | 10.57 (5.34, 15.80) | 55 | 10.96 (2.99, 18.93) | 56 | 9.73 (6.35, 13.12) |
| | B | 90 | 9.41 (7.45, 11.37) | 88 | 4.66 (3.78, 5.53) | 88 | 5.21 (4.22, 6.19)[a] |
| **3-HPMA (µg/24h)** | A | 54 | 1175.12 (1016.03, 1334.21) | 55 | 1212.50 (987.27, 1437.74) | 56 | 1160.45 (978.58, 1342.33) |
| | B | 90 | 1148.89 (1001.80, 1295.96) | 88 | 344.49 (283.22, 405.77) | 90 | 364.23 (307.65, 420.81) |
| **HMPMA (µg/24h)** | A | 54 | 440.17 (382.12, 498.22) | 55 | 395.41 (326.40, 464.43) | 56 | 376.12 (317.68, 434.55) |
| | B | 90 | 429.85 (384.96, 474.73) | 88 | 102.19 (86.93, 117.46) | 90 | 106.55 (88.48, 124.62) |
| **MHBMA (µg/24h)** | A | 54 | 4.11 (3.04, 5.18) | 55 | 4.16 (3.01, 5.30) | 56 | 3.70 (2.46, 4.95) |
| | B | 90 | 3.78 (3.10, 4.47) | 88 | 0.40 (0.27, 0.52) | 90 | 0.35 (0.25, 0.44) |
| **HEMA (µg/24h)** | A | 54 | 10.42 (7.24, 13.60) | 55 | 8.31 (6.22, 10.40) | 56 | 4.95 (3.79, 6.11) |
| | B | 90 | 8.90 (7.39, 10.40) | 88 | 4.71 (3.49, 5.94) | 90 | 1.38 (1.15, 1.61) |
| **4-ABP (ng/24h)** | A | 54 | 19.15 (16.64, 21.66) | 55 | 16.95 (14.31, 19.58) | 56 | 16.95 (13.95, 19.94) |
| | B | 90 | 18.32 (16.13, 20.50) | 88 | 4.18 (3.57, 4.79) | 90 | 3.96 (3.39, 4.52) |
| **2-AN (ng/24h)** | A | 54 | 27.18 (23.12, 31.24) | 55 | 24.62 (20.41, 28.83) | 56 | 25.76 (21.33, 30.19) |
| | B | 90 | 25.43 (22.37, 28.50) | 88 | 3.91 (2.48, 5.33) | 90 | 3.79 (2.53, 5.06) |
| ***o*-Tol** | A | 54 | 213.19 (97.78, 328.60) | 54 | 158.90 (130.34, 187.46)[b] | 56 | 155.66 (128.53, 182.78) |

| | | | | | | | |
|---|---|---|---|---|---|---|---|
| **(ng/24h)** | B | 90 | 176.96 (133.12, 220.79) | 88 | 56.34 (47.58, 65.09) | 90 | 59.09 (50.09, 68.10) |
| **1-OHP (ng/24h)** | A | 54 | 260.82 (212.99, 308.66) | 55 | 308.93 (250.88, 366.97) | 56 | 326.41 (245.94, 406.88) |
| | B | 90 | 251.27 (217.74, 284.80) | 88 | 127.78 (84.60, 170.95) | 90 | 168.75 (117.06, 220.44) |
| **eCO (ppm)** | A | 59 | 11.24 (9.02, 13.45) | 58 | 12.16 (10.19, 14.12) | 59 | 11.83 (9.65, 14.01) |
| | B | 97 | 9.34 (8.14, 10.54) | 97 | 1.39 (1.02, 1.76) | 96 | 1.76 (1.00, 2.52) |
| **TNeq (mg/24h)** | A | 54 | 18.14 (15.44, 20.83) | 55 | 16.03 (12.28, 19.78) | 56 | 13.37 (11.22, 15.53) |
| | B | 90 | 17.41 (15.05, 19.77) | 88 | 12.29 (10.71, 13.86) | 90 | 11.69 (10.26, 13.12) |
| **S-PMA (µg/24h)** | A | 54 | 4.55 (3.40, 5.69) | 55 | 3.87 (2.87, 4.86) | 56 | 4.08 (2.77, 5.39) |
| | B | 90 | 4.14 (3.48, 4.80) | 88 | 0.36 (0.23, 0.48) | 90 | 0.37 (0.25, 0.48) |
| **CEMA (µg/24h)** | A | 54 | 176.79 (152.82, 200.75) | 55 | 168.95 (141.71, 196.19) | 56 | 182.81 (154.77, 210.86) |
| | B | 90 | 183.84 (161.48, 206.19) | 88 | 15.92 (8.56, 23.28) | 90 | 12.48 (6.37, 18.59) |

Note: Group A, continue to smoke combustible cigarettes; Group B, switch to THP. Data are means (95% CI). N, number of participants; for details of participant composition refer to Supplementary Table 1. [a]Two outliers with values of 456.26 and 79.22 ng/24h were removed. [b]A single outlier with a value of 41,329 ng/24h was removed. NNAL, 4-(methylnitrosamino)-1-(3-pyridyl)-1-butanol; NNN, N-nitrosonornicotine; 3-HPMA, 3-hydroxypropylmercapturic acid; HMPMA, 3-hydroxy-1-methylpropylmercapturic acid; MHBMA, monohydroxybutenyl-mercapturic acid; HEMA, 2-hydroxyethylmercapturic acid; 4-ABP, 4-aminobiphenyl; 2-AN, 2-aminonaphthalene; *o*-Tol, *o*-Toluidine; 1-OHP, 1-hydroxypyrene; eCO, exhaled carbon monoxide; TNeq, total nicotine equivalents (nicotine, cotinine, 3-hydroxycotinine and their glucuronide conjugates); S-PMA, S-phenylmercapturic acid; CEMA, 2-cyanoethylmercapturic acid.

**Supplementary Table 4. BoE Levels at Baseline (Day 1), Day 90 and Day 180 in the Per-Protocol Population**

| Biomarker (units) | Group | N | Day 1 | N | Day 90 | N | Day 180 |
|---|---|---|---|---|---|---|---|
| **Total NNAL (ng/24h)** | A | 57 | 215.27 (175.16, 255.39) | 59 | 204.29 (157.31, 251.27) | 56 | 221.94 (177.86, 266.02) |
| | B | 123 | 204.53 (176.35, 232.71) | 126 | 92.86 (76.59, 109.13) | 117 | 115.70 (99.37, 132.03) |
| | D | 117 | 232.41 (203.92, 260.91) | 124 | 39.87 (30.40, 49.33) | 107 | 59.09 (39.41, 78.78) |
| | E | 33 | 12.80 (7.61, 17.98) | 36 | 6.44 (3.10, 9.78) | 33 | 11.54 (5.95, 17.13) |
| **Total NNN (ng/24h)** | A | 57 | 10.25 (5.29, 15.21) | 59 | 13.18 (4.63, 21.73) | 56 | 9.73 (6.35, 13.12) |
| | B | 122 | 9.68 (8.05, 11.31)[a] | 126 | 5.04 (3.92, 6.16) | 115 | 6.05 (4.52, 7.59)[b] |
| | D | 116 | 10.70 (7.95, 13.45)[c] | 124 | 5.86 (2.21, 9.51) | 106 | 2.94 (2.13, 3.76)[d] |
| | E | 33 | 1.44 (0.33, 2.54) | 36 | 1.18 (0.60, 1.76) | 33 | 1.24 (0.60, 1.89) |
| **3-HPMA (µg/24h)** | A | 57 | 1152.95 (998.07, 1307.84) | 59 | 1238.73 (1015.07, 1462.40) | 56 | 1160.45 (978.58, 1342.33) |
| | B | 123 | 1240.63 (1078.53, 1402.74) | 126 | 468.84 (373.50, 564.18) | 117 | 442.94 (368.11, 517.78) |
| | D | 117 | 1232.16 (1096.14, 1368.17) | 124 | 347.24 (293.84, 400.65) | 107 | 351.99 (275.49, 428.48) |
| | E | 33 | 288.50 (184.02, 392.98) | 36 | 281.66 (126.50, 436.82) | 33 | 261.83 (166.22, 357.45) |
| **HMPMA (µg/24h)** | A | 57 | 437.60 (380.94, 494.25) | 59 | 400.83 (332.89, 468.77) | 56 | 376.12 (317.68, 434.55) |
| | B | 123 | 455.11 (409.93, 500.30) | 126 | 136.63 (114.78, 158.49) | 117 | 133.09 (111.06, 155.12) |
| | D | 117 | 427.72 (385.27, 470.16) | 124 | 119.96 (100.49, 139.43) | 107 | 106.62 (90.91, 122.33) |
| | E | 33 | 111.00 (73.71, 148.28) | 36 | 96.72 (67.81, 125.64) | 33 | 81.55 (66.29, 96.82) |
| **MHBMA (µg/24h)** | A | 57 | 4.10 (3.07, 5.13) | 59 | 4.09 (3.00, 5.17) | 56 | 3.70 (2.46, 4.95) |
| | B | 123 | 4.02 (3.40, 4.64) | 126 | 0.96 (0.63, 1.29) | 117 | 0.69 (0.45, 0.93) |
| | D | 117 | 3.85 (3.14, 4.56) | 124 | 0.40 (0.20, 0.60) | 107 | 0.47 (0.28, 0.67) |
| | E | 33 | 0.09 (0.01, 0.16) | 36 | 0.15 (0.09, 0.22) | 33 | 0.24 (0.17, 0.32) |
| **HEMA (µg/24h)** | A | 57 | 10.06 (7.02, 13.09) | 59 | 8.11 (6.12, 10.09) | 56 | 4.95 (3.79, 6.11) |
| | B | 123 | 9.11 (7.85, 10.37) | 126 | 5.22 (4.17, 6.28) | 117 | 1.79 (1.43, 2.15) |
| | D | 117 | 10.97 (9.48, 12.47) | 124 | 7.70 (5.70, 9.69) | 107 | 1.79 (1.40, 2.18) |
| | E | 33 | 4.75 (3.58, 5.92) | 36 | 4.64 (2.05, 7.23) | 33 | 1.30 (0.98, 1.61) |

| | | | | | | | |
|---|---|---|---|---|---|---|---|
| **4-ABP (ng/24h)** | A | 57 | 18.95 (16.52, 21.38) | 59 | 17.16 (14.51, 19.81) | 56 | 16.95 (13.95, 19.94) |
| | B | 123 | 19.48 (16.99, 21.97) | 126 | 6.16 (4.96, 7.36) | 117 | 5.06 (4.31, 5.82) |
| | D | 117 | 20.37 (18.27, 22.47) | 124 | 5.53 (4.67, 6.39) | 107 | 5.12 (4.27, 5.98) |
| | E | 33 | 3.74 (3.09, 4.39) | 36 | 3.28 (2.12, 4.44) | 33 | 3.25 (2.51, 4.00) |
| **2-AN (ng/24h)** | A | 57 | 26.83 (22.90, 30.77) | 59 | 25.27 (20.88, 29.66) | 56 | 25.76 (21.33, 30.19) |
| | B | 123 | 27.87 (24.33, 31.42) | 126 | 6.37 (4.53, 8.21) | 117 | 5.95 (4.36, 7.54) |
| | D | 117 | 28.29 (25.03, 31.54) | 124 | 4.35 (3.29, 5.42) | 107 | 4.35 (3.25, 5.45) |
| | E | 33 | 1.89 (1.48, 2.30) | 36 | 1.70 (1.04, 2.36) | 33 | 2.27 (1.34, 3.20) |
| ***o*-Tol (ng/24h)** | A | 57 | 210.25 (100.93, 319.56) | 58 | 159.04 (131.92, 186.16)[e] | 56 | 155.65 (128.53, 182.78) |
| | B | 123 | 182.22 (147.36, 217.08) | 126 | 69.56 (59.54, 79.58) | 117 | 64.79 (56.56, 73.02) |
| | D | 117 | 176.33 (157.40, 195.27) | 124 | 92.66 (60.39, 124.92) | 106 | 73.63 (50.77, 96.48)[f] |
| | E | 33 | 48.99 (40.16, 57.81) | 36 | 39.63 (33.42, 45.84) | 33 | 40.97 (32.94, 49.00) |
| **1-OHP (ng/24h)** | A | 57 | 263.73 (217.36, 310.10) | 59 | 304.55 (249.25, 359.85) | 56 | 326.41 (245.93, 406.88) |
| | B | 123 | 286.25 (231.38, 341.13) | 126 | 141.00 (108.76, 173.24) | 116 | 160.39 (125.08, 195.70)[g] |
| | D | 117 | 378.32 (322.62, 434.03) | 124 | 213.75 (142.28, 285.22) | 107 | 136.26 (109.83, 162.69) |
| | E | 33 | 116.41 (91.58, 141.25) | 36 | 123.38 (57.86, 188.90) | 33 | 77.98 (64.08, 91.88) |
| **eCO (ppm)** | A | 63 | 11.1 (9.0, 13.2) | 62 | 12.4 (10.4, 14.3) | 59 | 11.8 (9.7, 14.0) |
| | B | 140 | 10.0 (8.9, 11.1) | 139 | 2.2 (1.6, 2.8) | 126 | 2.4 (1.6, 3.1) |
| | D | 136 | 11.5 (10.4, 12.7) | 134 | 1.8 (1.4, 2.1) | 108 | 1.4 (1.1, 1.7) |
| | E | 37 | 0.9 (0.6, 1.1) | 37 | 0.7 (0.4, 1.0) | 37 | 1.1 (0.9, 1.3) |
| **TNeq (mg/24h)** | A | 57 | 17.88 (15.25, 20.51) | 59 | 16.12 (12.49, 19.76) | 56 | 13.37 (11.22, 15.53) |
| | B | 123 | 17.92 (15.91, 19.94) | 126 | 12.75 (11.31, 14.18) | 117 | 11.80 (10.55, 13.04) |
| | D | 117 | 14.85 (13.22, 16.49) | 124 | 2.93 (2.12, 3.75) | 107 | 2.52 (1.53, 3.50) |
| | E | 33 | 0.03 (0.01, 0.04) | 36 | 0.01 (0.01, 0.01) | 33 | 0.01 (0.00, 0.01) |
| **S-PMA (µg/24h)** | A | 57 | 4.54 (3.44, 5.64) | 59 | 3.88 (2.91, 4.85) | 56 | 4.08 (2.77, 5.39) |
| | B | 123 | 4.41 (3.82, 5.01) | 126 | 0.91 (0.59, 1.23) | 117 | 0.74 (0.51, 0.97) |
| | D | 117 | 4.36 (3.75, 4.98) | 124 | 0.64 (0.41, 0.86) | 107 | 0.56 (0.34, 0.77) |
| | E | 33 | 0.28 (0.20, 0.36) | 36 | 0.25 (0.21, 0.28) | 33 | 0.19 (0.15, 0.23) |

| | | | | | | | |
|---|---|---|---|---|---|---|---|
| **CEMA (µg/24h)** | A | 57 | 175.46 (152.03, 198.88) | 59 | 173.56 (144.38, 202.75) | 56 | 182.81 (154.77, 210.86) |
| | B | 123 | 194.52 (172.57, 216.47) | 126 | 35.80 (25.04, 46.55) | 117 | 31.67 (20.88,42.46) |
| | D | 117 | 181.62 (160.63, 202.62) | 124 | 20.92 (13.69, 28.14) | 107 | 23.86 (13.69, 34.03) |
| | E | 33 | 1.88 (1.59, 2.16) | 36 | 1.75 (1.42, 2.07) | 33 | 1.78 (1.42, 2.14) |

Note: Group A, continue to smoke combustible cigarettes; Group B, switch to THP; Group D, cessation; Group E, never-smokers. Data are means (95% CI). N, number of participants; for details of participant composition refer to Supplementary Table 1. [a]A single outlier with a value of 1015.16 ng/24h was removed. [b]Two outliers with values of 636.06 and 456.26 ng/24h were removed. [c]A single outlier with a value of 349.72 ng/24h was removed. [d]A single outlier with a value of 297.65 ng/24h was removed. [e]A single outlier with a value of 41,329 ng/24h was removed. [f]A single outlier with a value of 16,989 ng/24h was removed. [g]A single outlier with a value of 27,972 ng/24h was removed. NNAL, 4-(methylnitrosamino)-1-(3-pyridyl)-1-butanol; NNN, N-nitrosonornicotine; 3-HPMA, 3-hydroxypropylmercapturic acid; HMPMA, 3-hydroxy-1-methylpropylmercapturic acid; MHBMA, monohydroxybutenyl-mercapturic acid; HEMA, 2-hydroxyethylmercapturic acid; 4-ABP, 4-aminobiphenyl; 2-AN, 2-aminonaphthalene; *o*-Tol, *o*-Toluidine; 1-OHP, 1-hydroxypyrene; eCO, exhaled carbon monoxide; TNeq, total nicotine equivalents (nicotine, cotinine, 3-hydroxycotinine and their glucuronide conjugates); S-PMA, S-phenylmercapturic acid; CEMA, 2-cyanoethylmercapturic acid.

**Supplementary Table 5. Between-Group Statistical Analysis of Change from Baseline in BoE and BoPH in the Per-Protocol Population**

| Biomarker (units) | Group | N | Day | LS mean or GLS mean ratio compared to baseline[a] | Difference B-A or Ratio B:A (CI)[a] | *p* value |
|---|---|---|---|---|---|---|
| **Total NNAL (ng/24h)** | A | 55 | 90 | 0.88 | 0.51 (0.30, 0.85) | <0.0001 |
| | B | 114 | | 0.45 | | |
| | D | 108 | | 0.12 | - | - |
| **Total NNN (ng/24h)** | A | 55 | 90 | 0.82 | 0.50 (0.22, 1.12) | 0.00306 |
| | B | 114 | | 0.41 | | |
| | D | 108 | | 0.27 | - | - |
| **Total NNN (ng/24h)** | A | 53 | 180 | 1.03 | 0.63 (0.36, 1.11) | 0.02758 |
| | B | 106 | | 0.65 | | |
| | D | 96 | | 0.23 | - | - |
| **3-HPMA (µg/24h)** | A | 55 | 90 | 0.98 | 0.32 (0.22, 0.47) | <0.00004 |
| | B | 114 | | 0.32 | | |
| | D | 108 | | 0.28 | - | - |
| **HMPMA (µg/24h)** | A | 55 | 90 | 0.84 | 0.32 (0.21, 0.47) | <0.00004 |
| | B | 114 | | 0.27 | | |
| | D | 108 | | 0.23 | - | - |
| **MHBMA (µg/24h)** | A | 55 | 90 | 1.22 | 0.13 (0.06, 0.27) | <0.00004 |
| | B | 114 | | 0.16 | | |
| | D | 108 | | 0.07 | - | - |
| **HEMA (µg/24h)** | A | 55 | 90 | 0.81 | 0.52 (0.33, 0.83) | <0.00004 |
| | B | 114 | | 0.42 | | |
| | D | 108 | | 0.60 | - | - |

| | | | | | | |
|---|---|---|---|---|---|---|
| **4-ABP (ng/24h)** | A | 55 | 90 | 0.86 | 0.30 (0.21, 0.44) | <0.00004 |
| | B | 114 | | 0.26 | | |
| | D | 108 | | 0.26 | - | - |
| **2-AN (ng/24h)** | A | 55 | 90 | 0.88 | 0.17 (0.10, 0.29) | <0.00004 |
| | B | 114 | | 0.15 | | |
| | D | 108 | | 0.12 | - | - |
| ***o*-Tol (ng/24h)** | A | 55 | 90 | 1.00 | 0.37 (0.25, 0.54) | <0.00004 |
| | B | 114 | | 0.37 | | |
| | D | 108 | | 0.40 | - | - |
| **1-OHP (ng/24h)** | A | 55 | 90 | 1.07 | 0.38 (0.25, 0.60) | <0.00004 |
| | B | 114 | | 0.41 | | |
| | D | 108 | | 0.48 | - | - |
| **FeNO (ppb)** | A | 54 | 180 | 1.05 | 1.39 (1.12, 1.74) | <0.0001 |
| | B | 121 | | 1.46 | | |
| | D | 99 | | 1.80 | - | - |
| **WBC count ($10^9$/L)** | A | 56 | 180 | 0.98 | 0.89 (0.81, 0.97) | 0.00045 |
| | B | 120 | | 0.87 | | |
| | D | 100 | | 0.89 | - | - |
| **eCO (ppm)** | A | 62 | 120/150 | 15.06 | -12.54 (-15.52, -9.57) | <0.00004 |
| | B | 136 | | 2.51 | | |
| | D | ND | | ND | - | - |
| **TNeq (mg/24h)** | A | 55 | 90 | -0.95 | -3.42 (-7.66, 0.82) | 0.00549 |
| | B | 114 | | -4.37 | | |
| | D | 108 | | -12.84 | - | - |
| **TNeq (mg/24h)** | A | 53 | 180 | -3.55 | -1.87 (-4.38, 0.64) | 0.04639 |
| | B | 106 | | -5.42 | | |

| | | | | | | |
|---|---|---|---|---|---|---|
| | D | 96 | | -13.59 | - | - |
| **S-PMA (µg/24h)** | A | 55 | 90 | -0.66 | -2.86 (-3.91, -1.82) | <0.00004 |
| | B | 114 | | -3.53 | | |
| | D | 108 | | -3.70 | - | - |
| **CEMA (µg/24h)** | A | 55 | 90 | -13 | -141 (-176, -106) | <0.00004 |
| | B | 114 | | -154 | | |
| | D | 108 | | -162 | - | - |
| **11-dTx B2 (ng/24h)** | A | 53 | 180 | -141 | -201 (-402, 0) | 0.00744 |
| | B | 106 | | -343 | | |
| | D | 96 | | -249 | - | - |
| **8-Epi-PGF2α (ng/24h)** | A | 53 | 180 | -45 | -70 (-125, -15) | 0.0007 |
| | B | 106 | | -115 | | |
| | D | 96 | | -82 | - | - |

Note: Group A, continue to smoke combustible cigarettes; Group B, switch to THP; Group D, cessation. Day 90 analyses were performed on the D90 PP population, while day 180 analyses were performed on the D180 PP population. All analyses, except for eCO, were performed using biomarker levels at baseline (day 1) and on either day 90 or day 180, as indicated. eCO was analysed as the difference between the means of absolute values on days 120 and 150. LS, least squares; GLS, geometric least squares; N, number of participants, for details of participant composition refer to Supplementary Table 1; ND, not determined; CI, confidence interval; 99.94% CI shown for day 90; 99.245% CI shown for day 180. Significance level = 0.0006 on day 90 and 0.00755 on day 180. [a]GLS mean and ratio shown for data log-transformed prior to calculation of change from baseline: NNAL, 4-(methylnitrosamino)-1-(3-pyridyl)-1-butanol; NNN, N-nitrosonornicotine; 3-HPMA, 3-hydroxypropylmercapturic acid; HMPMA, 3-hydroxy-1-methylpropylmercapturic acid; MHBMA, monohydroxybutenyl-mercapturic acid; HEMA, 2-hydroxyethylmercapturic acid; 4-ABP, 4-aminobiphenyl; 2-AN, 2-aminonaphthalene; *o*-Tol, *o*-Toluidine; 1-OHP, 1-hydroxypyrene; FeNO, fractional exhaled nitric oxide; WBC, white blood cell. LS mean and difference shown for untransformed data: eCO, exhaled carbon monoxide; TNeq, total nicotine equivalents (nicotine, cotinine, 3-hydroxycotinine and their glucuronide conjugates); S-PMA, S-phenylmercapturic acid; CEMA, 2-cyanoethylmercapturic acid; 11-dTx B2, 11-dehydrothromboxane B2; 8-Epi-PGF2α, 8-epi-Prostaglandin F2α type III.

**Supplementary Table 6. Descriptive Statistics for sICAM-1, HDL and $FEV_1$ at Baseline (day 1), Day 90 and Day 180 in the Per-Protocol Population**

| Biomarker (units) | Group | N | Day 1 | Day 90 | Day 180 |
|---|---|---|---|---|---|
| sICAM-1 (ng/mL) | A | 59-63 | 468.4 (440.0, 496.7) | 485.8 (452.0, 519.5) | 501.8 (463.6, 540.0) |
| | B | 126-139 | 477.2 (453.7, 500.8) | 442.3 (422.1, 462.5) | 455.6 (430.7, 480.6) |
| | D | 108-133 | 426.0 (405.2, 446.8) | 395.5 (381.0, 410.0) | 415.8 (397.7, 433.9) |
| | E | 37 | 350.7 (324.9, 376.6) | 348.9 (323.0, 374.8) | 415.5 (351.9, 479.2) |
| HDL (mmol/L) | A | 59-63 | 1.39 (1.29, 1.49) | 1.39 (1.29, 1.50) | 1.37 (1.26, 1.49) |
| | B | 126-140 | 1.41 (1.35, 1.47) | 1.47 (1.41, 1.54) | 1.47 (1.40, 1.54) |
| | D | 108-135 | 1.51 (1.45, 1.57) | 1.61 (1.53, 1.68) | 1.54 (1.47, 1.60) |
| | E | 37 | 1.68 (1.55, 1.82) | 1.71 (1.59, 1.84) | 1.72 (1.58, 1.86) |
| $FEV_1$%pred | A | 55-62 | 91.1 (88.2, 94.0) | 90.0 (87.1, 92.9) | 88.1 (85.1, 91.0) |
| | B | 122-133 | 91.1 (89.2, 93.0) | 91.6 (89.6, 93.5) | 91.7 (89.6, 93.7) |
| | D | 100-132 | 93.8 (91.9, 95.7) | 94.4 (92.6, 96.3) | 92.1 (89.9, 94.3) |
| | E | 34-35 | 100.2 (96.5, 103.9) | 100.4 (96.6, 104.1) | 98.0 (94.2, 101.8) |

Note: Data shown are means with lower and upper 95% confidence intervals in parentheses and include data to day 90 for participants in the D90 PP population who were not in the D180 PP population, and all data for participants in the D180 PP population. N, number of participants; for details of participant composition refer to Supplementary Table 1. Group A, continue to smoke combustible cigarettes; Group B, switch to THP; Group D, cessation; Group E, nevers-smokers. sICAM-1, soluble intercellular adhesion molecule-1; HDL, high-density lipoprotein; $FEV_1$%pred, forced expiratory volume in 1 second percentage of predicted.
